# Crowdsourcing and the Accuracy of Online Information Regarding Weight Gain in Pregnancy: A Descriptive Study

**DOI:** 10.2196/jmir.5138

**Published:** 2016-04-07

**Authors:** Tammy Chang, Bianca A Verma, Trevor Shull, Michelle H Moniz, Lauren Kohatsu, Melissa A Plegue, Kevyn Collins-Thompson

**Affiliations:** ^1^ Department of Family Medicine University of Michigan Ann Arbor, MI United States; ^2^ Institute for Healthcare Policy and Innovation University of Michigan Ann Arbor, MI United States; ^3^ University of Michigan Medical School Ann Arbor, MI United States; ^4^ Department of Obstetrics & Gynecology University of Michigan Ann Arbor, MI United States; ^5^ Optimity Advisors Washington, DC United States; ^6^ School of Information University of Michigan Ann Arbor, MI United States

**Keywords:** Internet, crowdsourcing, weight gain, pregnancy

## Abstract

**Background:**

Excess weight gain affects nearly half of all pregnancies in the United States and is a strong risk factor for adverse maternal and fetal outcomes, including long-term obesity. The Internet is a prominent source of information during pregnancy; however, the accuracy of this online information is unknown.

**Objective:**

To identify, characterize, and assess the accuracy of frequently accessed webpages containing information about weight gain during pregnancy.

**Methods:**

A descriptive study was used to identify and search frequently used phrases related to weight gain during pregnancy on the Google search engine. The first 10 webpages of each query were characterized by type and then assessed for accuracy and completeness, as compared to Institute of Medicine guidelines, using crowdsourcing.

**Results:**

A total of 114 queries were searched, yielding 305 unique webpages. Of these webpages, 181 (59.3%) included information regarding weight gain during pregnancy. Out of 181 webpages, 62 (34.3%) contained no specific recommendations, 48 (26.5%) contained accurate but incomplete recommendations, 41 (22.7%) contained complete and accurate recommendations, and 22 (12.2%) were inaccurate. Webpages were most commonly from for-profit websites (112/181, 61.9%), followed by government (19/181, 10.5%), medical organizations or associations (13/181, 7.2%), and news sites (12/181, 6.6%). The largest proportion of for-profit sites contained no specific recommendations (44/112, 39.3%). Among pages that provided inaccurate information (22/181, 12.2%), 68% (15/22) were from for-profit sites.

**Conclusions:**

For-profit websites dominate the online space with regard to weight gain during pregnancy and largely contain incomplete, inaccurate, or no specific recommendations. This represents a significant information gap regarding an important risk factor for obesity among mothers and infants. Our findings suggest that greater clinical and public health efforts to disseminate accurate information regarding healthy weight gain during pregnancy may help prevent significant morbidity and may support healthier pregnancies among at-risk women and children.

## Introduction

Appropriate weight gain during pregnancy has important health implications for both mothers and infants [1]. Excess weight gain in pregnancy is associated with adverse obstetric and fetal/neonatal outcomes, including hypertensive disorders of pregnancy, gestational diabetes, excessive fetal growth, prolonged labor, birth injury, and cesarean delivery [2,3]. Furthermore, excess weight gain in pregnancy may be associated with long-term increased risk of obesity in both mother and child [4-7].

In 2009, the Institute of Medicine (IOM) updated its guidelines for weight gain in pregnancy, recommending more stringent weight gain with increasing prepregnancy body mass index (BMI) [1]. However, many women are unaware of these guidelines [8,9]. In the United States, only 32% of women gain the appropriate amount of weight during pregnancy based on the IOM guidelines, while 47% of women gain excessive weight and 21% gain inadequate weight [10]. Although the American Congress of Obstetricians and Gynecologists (ACOG) recommends discussing appropriate weight gain, diet, and exercise at the initial visit and periodically throughout pregnancy, clinicians caring for pregnant women often do not follow this recommendation [11,12]. In one recent study of over 300 women, only 12% reported being counseled correctly by their health care provider regarding how much they should gain during pregnancy [11]. Clinicians may be unaware, unfamiliar, or unaccepting of the guidelines, or reluctant to discuss the sensitive topic of weight gain with their overweight or obese patients [13-15].

The Internet is a common source of health information among pregnant women, with nearly all women (94%) using the Internet for pregnancy-related information [16-19]. Among these women, nearly all (98%) begin their online health inquiries using search engines such as Google [18]. Most women (99%) search online because they want to find out more information on their own, beyond the information that is provided to them by their health care provider [18]. Pregnant women commonly seek information about topics such as antenatal complications, childbirth, pregnancy symptoms, and health promotion/lifestyle issues, including nutrition and physical activity during pregnancy [18-20]. In general, pregnant women consider online health information to be of reasonable quality and reliable, and information found online plays a significant role in women’s decision-making during pregnancy [17-19].

Despite widespread online health information seeking during pregnancy, relatively little is known about whether online sources accurately reflect the latest IOM guidelines for weight gain in pregnancy. The objective of this study was to characterize the type of website and accuracy of information of the most frequently accessed websites containing information about weight gain in pregnancy.

## Methods

### Identification of Commonly Accessed Webpages

The authors identified 13 initial search queries that included keywords and phrases related to weight gain during pregnancy based on clinical experiences with pregnant patients (ie, “Am I gaining enough weight during pregnancy?,” “Healthy weight gain during pregnancy,” and “Am I gaining too much weight during pregnancy?”). To help ensure the queries used by our study reflected how people actually search on this topic, we expanded the initial set of 13 queries into 114 variants based on query suggestions produced by Google’s autocomplete feature (see Multimedia Appendix 1). The autocomplete feature suggests similar queries that have been typed before by Google users, or that occur as content on the Web [21].

Each query was entered into the Google search engine to yield a list of webpages on August 8, 2014. A website is defined as a set of webpages typically served from a single Web domain [22]. A Windows 8 Pro (64-bit) machine using the US English locale and running Google Chrome (v46) from Ann Arbor, Michigan, was used for all search results retrieval and processing. A new Google account was used with personalization not activated, so that search history did not influence the results. Safe search was also not activated.

The first 10 webpages that resulted from each search were included in this study, as these represent the websites most likely to be viewed by actual users; webpages ranked 11 or greater on a Google search received less than 5% of overall traffic [23]. Paid advertisements/webpages at the top of the Google search result were not included. Duplicate websites were removed to create the list of the most commonly accessed websites resulting from Google searching for information on weight gain during pregnancy.

### Data Collection

#### Crowdsourcing as Data Collection Method

Crowdsourcing was used in our study to characterize the type and accuracy of the commonly accessed webpages. Crowdsourcing is the process of “obtaining needed services, ideas, or content by soliciting contributions from a large group of people, and especially from the online community, rather than from traditional employees or suppliers” [24]. This online data collection method is increasingly used in health-related research and is an ideal method for this study for two reasons: (1) crowdsourcing obtains the judgments of individuals who are likely more similar to patients than medical experts and (2) crowdsourcing employs several layers of validation to ensure a high level of accuracy in data collection [25-28]. In our case, Crowdflower.com [29] was used to identify US residents to complete *microtasks*, or small tasks online that involved identifying simple ideas or phrases from webpages. People who complete microtasks online are called contributors and receive a small reimbursement for the tasks they complete. Contributors were instructed to evaluate only the body of the webpage (“corpus”) and to disregard any advertisements or pop-ups. Contributors were paid between US $0.05 and $0.15 for each microtask completed, depending on the difficulty of the task.

#### Crowdflower Tasks

Each webpage was analyzed independently by three Crowdflower contributors. Contributors were drawn from the pool of millions of Crowdflower contributors and webpages were assigned randomly. Thus, the three contributors assigned to each webpage varied. Each of the following three questions was a separate *task* used to evaluate each of the webpages:

1. Task 1: Is the webpage about weight gain during pregnancy?

2. Task 2: What type of website is this (eg, for-profit company, government, news, medical organization, blog, university, nonprofit/foundation, or medical journal)?

3. Task 3: Did this webpage include the Institute of Medicine guidelines information for total weight gain for each prepregnancy weight group [1]?

These three tasks were completed between September 20, 2014, and December 3, 2014. Textbox 1 contains the abstraction questionnaire with the exact questions and answer choices used by contributors to evaluate each webpage.

**Abstraction questionnaire, including questions and answer choices.**

Task 1: Does this webpage include information about healthy weight gain DURING pregnancy?

- Yes
- No

Task 2: Which description below best describes the type of webpage this is?

- Government (.gov)
- News (primary goal of site is to report news)
- University or academic center with associated hospitals/clinics (examples: Mayo Clinic, Kaiser Permanente)
- Company (for-profit business, examples: babycenter.com, WebMD)
- Blog
- Medical organization or associations (examples: Institute of Medicine [IOM], the American College of Obstetricians and Gynecologists [ACOG])
- Medical journal (example: American Journal of Obstetrics and Gynecology)
- Nonprofit/foundation (example: March of Dimes)
- Other

Task 3: Which of the following SPECIFIC recommendations for TOTAL weight gain during pregnancy were mentioned, if any? Select all that apply.

- Underweight women (or women with body mass index [BMI] less than 18.5 kg/m^2^) are recommended to gain 28-40 pounds
- Normal-weight women (or women with BMI 18.5-24.9 kg/m^2^) are recommended to gain 25-35 pounds
- Overweight women (or women with BMI 25-29.9 kg/m^2^) are recommended to gain 15-25 pounds
- Obese women (or women with BMI ≥30 kg/m^2^) are recommended to gain 11-20 pounds
- Recommendations were given in pounds (lbs), but were different than what is listed above for one or more groups of women
- Recommendations were only given in kilograms (kg)
- No SPECIFIC recommendations for total weight gain during pregnancy were given
- No SPECIFIC recommendations for total weight gain during pregnancy were given in the text, though there is an ONLINE CALCULATOR
- Recommendations were only given for multiple pregnancies (twins)

#### Quality Control of Crowdflower Responses

The authors used three methods to ensure the accuracy of contributors’ work. First, Crowdflower itself provides a quality-control mechanism by maintaining historical trust levels for each contributor, based on their performance from previous jobs. These trust levels are used to maintain a high-quality pool of potential contributors. Second, before they could contribute to the study, potential contributors were required to successfully complete a training session in which they correctly evaluated at least two of three gold standard test webpages. These test webpages were a subset of the total webpages, for which gold standard responses to the three abstraction form questions were previously agreed upon by two investigators (TC, TS, BV, or LK). Third, additional gold standard test webpages—unidentified to contributors—were interspersed randomly throughout the full sample of webpages for each task. Contributors were required to maintain at least 65% accuracy of these test questions to be considered *trusted* contributors. The process of determining accuracy was automated by Crowdflower and reported to contributors in real time. If a contributor’s accuracy decreased below this threshold, they were removed from the job and their prior responses were disregarded.

#### Task Flow for Crowdsourced Data Collection

Despite the use of specific search terms, some webpages were determined *not* to be about weight gain during pregnancy and were excluded from further analyses using the question in Task 1. For this task, if at least two of three contributors determined the webpage to be about weight gain in pregnancy, it was considered a relevant webpage for further analysis.

The remaining webpages that did include information about weight gain during pregnancy were then evaluated using questions in Tasks 2 and 3. For Task 2, if at least two of three contributors agreed on the website type, this was considered the correct answer. If three contributors answered differently in Task 2, the webpage was reviewed by two investigators (TC, TS, BV, or LK) to determine the correct website type.

For Task 3, each contributor could select more than one response and each response selected by at least two of three contributors was deemed correct. Two investigators (TC, TS, BV, or LK) also reviewed any webpages that had no majority consensus in order to determine a final answer. Weight gain recommendations that were reported in kilograms were evaluated for accuracy using the technique described above. Webpages that only included recommendations for multiple gestations (eg, twins) were removed from our sample (n=1).

#### Researcher Assessment of Crowdflower Results

Each webpage’s recommendations for total weight gain were classified as *complete and accurate* (consistent with IOM recommendations for each prepregnancy BMI category), *incomplete but accurate* (consistent with IOM recommendations but exclusive of one or more of the BMI categories), *inaccurate* (inconsistent with IOM recommendations, such as an incorrect range; recommendation listed was outside of the range; or incorrect BMI categories), *no recommendation* (no specific recommendations of weight gain ranges based on prepregnancy BMI), or *no recommendation but calculator* (for webpages that include a calculator to determine a user’s recommended weight gain, but do not include specific recommendations in the text of the page).

Inaccurate webpages were reviewed by one investigator (TC) to determine whether the webpage was reporting recommendations that were higher or lower than recommended by the IOM, or if the inaccuracy was related to errors or omission in reporting prepregnancy BMI ranges for their recommendation.

Descriptive statistics were used to describe the distribution of the *type of sites*, and the *accuracy* and *completeness* of webpages. This article was developed using publicly available information. No human participants took part in any protocol and, as such, this study was deemed exempt from review by the University of Michigan Institutional Review Board.

## Results

After querying the 114 search terms and aggregating the top 10 website results of each query and removing any duplicates, 305 unique webpages remained. Of these 305 unique webpages, 181 (59.3%) included information regarding weight gain during pregnancy. Webpages were most commonly from for-profit websites (112/181, 61.9%), followed by government (19/181, 10.5%), medical organizations or associations (13/181, 7.2%), and news sites (12/181, 6.6%) (see Table 1).

Overall, one-third (62/181, 34.3%) of all websites that contained information regarding weight gain during pregnancy gave no specific recommendations for total weight gain during pregnancy. The largest proportion of webpages overall were from for-profit sites that contained no specific recommendations (44/181, 24.3%). Among pages that provided inaccurate information (22/181, 12.2%), 68% (15/22) were from for-profit sites (see Table 2). Among pages that provided accurate and complete information (41/181, 22.7%), 41% (17/41) were from for-profit sites, 22% (9/41) from government sites, and 17% (7/41) from medical organizations. Please see Multimedia Appendix 2 for a list of the top 10 accurate webpages by frequency of appearance in Google searching.

**Table 1:** Frequency of each type of website (n=181).

Type of website	Frequency, n (%)
Company (for-profit business)	112 (61.9)
Government	19 (10.5)
Medical organization or association	13 (7.2)
News	12 (6.6)
Personal blog	10 (5.5)
Nonprofit/foundation	8 (4.4)
University or academic medical center	6 (3.3)
Medical journal	1 (0.6)

**Table 2:** Accuracy and completeness by type of website used; first page (top 10) results only (n=181^a^).

Type of domain/website of the selected webpages	Complete and accurate, n (%)	Incomplete but accurate, n (%)	Inaccurate, n (%)	No recommend- ation, n (%)	No recommend- ation, but weight gain calculator, n (%)
Government (n=19)	9 (47)	4 (21)	2 (11)	3 (16)	1 (5)
News (n=12)	4 (33)	4 (33)	0 (0)	4 (33)	0 (0)
Personal blog (n=10)	0 (0)	2 (20)	2 (20)	6 (60)	0 (0)
University or academic medical center (n=6)	2 (33)	2 (33)	0 (0)	2 (33)	0 (0)
Medical organization or association (n=13)	7 (54)	2 (15)	1 (8)	2 (15)	1 (8)
Nonprofit/foundation (n=8)	2 (25)	4 (50)	2 (25)	0 (0)	0 (0)
Company (for-profit business) (n=112)	17 (15.2)	30 (26.8)	15 (13.4)	44 (39.3)	6 (5.4)
Medical journal (n=1)	0 (0)	0 (0)	0 (0)	1 (100)	0 (0)
Overall (n=181)	41 (22.7)	48 (26.5)	22 (12.2)	62 (34.3)	8 (4.4)

^a^One site excluded for providing recommendations for twins.

Of inaccurate webpages, 18% (4/22) recommended more than the recommended amount of weight, 14% (3/22) recommended less, and 64% (14/22) of webpages included errors or omissions in reporting prepregnancy BMI ranges for their recommendations. The 4 pages out of 22 (18%) that recommended more weight gain reported between 2 and 5 pounds more than the recommended amount. For example, askdrsears.com [30] reported, “If you begin pregnancy slightly above your ideal weight, a healthy weight gain is 20 to 25 pounds; if you are obese, less than 20 pounds,” while the correct recommendation for overweight women is 15-25 pounds. For the 3 pages out of 22 (14%) that recommended less weight gain, they ranged from 1 to 8 pounds less. For example, sharecare.com [31] reported, “The recommended weight gain is 25-30 lbs for average weight women. 15-20 lbs if overweight.” Again, the recommendation for overweight women is 15-25 pounds. Among the webpages that included errors or omissions in reporting BMI ranges, errors in the prepregnancy BMI category deviated from the correct definition by 1-2 BMI points (kg/m^2^). Out of 22 websites, 4 (18%) combined the overweight and obese groups and combined the recommendations (ie, “Women with pre-pregnancy BMI >25 can gain between 11-25 pounds”).

## Discussion

### Principal Findings

The majority of webpages assessed in our study do not reflect the latest IOM guidelines and either present inaccurate or incomplete information, or provide no recommendations. Additionally, for-profit websites currently dominate this online sphere, and these were most likely to contain inaccurate, incomplete, or no specific recommendations compared to other website types. Our study suggests that despite established guidelines for healthy weight gain in pregnancy, many women may not access these guidelines when searching the Internet, potentially increasing their risk of adverse maternal or fetal outcomes from too much or too little weight gain.

Among inaccurate webpages, most recommendations either were within a few pounds of the IOM recommendations or incorrectly defined prepregnancy BMI categories by just 1-2 points (kg/m^2^). However, some webpages combined recommendations for overweight and obese women. This is concerning because obese women may be at highest risk of obstetric complications from excessive gestational weight gain. More concerning may be that nearly one-third of webpages had no recommendations despite appearing in the top 10 results of a Google query for information regarding weight gain during pregnancy. This creates a significant knowledge gap for pregnant women and can have long-term health consequences for mother and baby.

Patients may receive no or inaccurate information on weight gain in pregnancy not just from the Internet, but also from their clinicians, friends, and family. Studies have shown that patients want specific information on weight gain during pregnancy, and they trust and rely on information given by clinicians [32,33]. Clinicians also have the advantage of providing face-to-face information to their patients. However, clinicians are often incomplete information sources regarding weight gain during pregnancy because they fail to address the topic or provide information that is not aligned with the IOM guidelines [34,35]. Perhaps the same reluctance among clinicians to discuss the sensitive subject of weight may also apply to online contributors who may not want to offend their pregnant readers by discussing specific weight gain guidelines on their websites. This lack of information, from both clinicians and the Internet, may be compounded by misinformation, such as the widespread cultural belief of “eating for two,” that is often perpetuated and reinforced by pregnant women’s friends and family members [36-39].

Our findings underscore the inadequacy of online resources—key sources of health information for pregnant women—in addressing weight gain in pregnancy [10]. These findings also suggest two specific ways to improve awareness of the IOM weight gain guidelines, for both pregnant women and the people who influence them:

1. Clinicians and caregivers need to fill the knowledge gap. At this time, clinicians and those that provide support and care for pregnant women cannot depend on the Internet to transmit accurate information about healthy weight gain during pregnancy. As a trusted and preferred source of health information, clinicians, nurses, and public health workers should anticipate a knowledge gap about this topic and strive to discuss healthy weight gain with every pregnant woman [32,33]. Robust data from systematic reviews and meta-analyses have demonstrated the efficacy of motivational interviewing, behavioral self-monitoring, goal setting, structured moderate physical exercise programs, and dietary counseling [40-44]. Ideally, providers of prenatal care should address healthy diet and exercise habits early in pregnancy, including discussions of individualized goals for total weight gain across the pregnancy. The frequency of prenatal visits allows for revisiting this topic and drawing on clinical support resources, such as nurses, nutritional counselors, and social workers, to augment the clinician’s efforts.

In addition, because of the known challenges in patient-clinician communication regarding weight gain during pregnancy, research should also investigate effective communication approaches that work for both clinicians and at-risk patients. These approaches could include how the topic should be framed, what information should be presented, and the optimal timing of this counseling.

2. Accurate webpages must be more accessible. For-profit webpages dominate online search results because they know how to market their websites. Academic and government websites, on the other hand, do not seem to be using the techniques that ensure a webpage appears as a top result in Google searching. These techniques include optimizing the keywords used in the website, so that they are in the file name and headings and are used throughout the page, especially in the beginning of the first sentence; creating sitemaps (ie, a list of pages of a website accessible to crawlers or users); and including related links that can be detected and indexed to create greater visibility for the website and the health message [45]. By employing these techniques, websites with accurate information can become more accessible by appearing higher in Google searching, something shown to improve Web traffic to those sites [23]. These techniques can have an exponential effect as other sites can link to them and spread the accurate and usable knowledge. Furthermore, visible and easily searchable sites can help to educate not only pregnant women, but other influential people, such as family and friends who often search on behalf of their friends and loved ones [46].

### Limitations

This study describes the text that is contained in the webpage, not whether a patient understood the text, as we did not evaluate the presentation or readability of the information. Also, the specific characteristics and motivations of contributors on Crowdflower are unknown and may bias their judgments, though we believe this bias is limited by the multiple layers of quality control described in the Methods. We also did not analyze the accuracy of online calculators, though they were a small proportion of webpages (8/181, 4.4%). Finally, only the Google search engine results were evaluated in this study, which may limit the generalizability of our findings.

### Conclusions

In conclusion, frequently accessed online information regarding weight gain during pregnancy does not reflect the latest IOM guidelines, with a large proportion of webpages displaying inaccurate or incomplete information, or no recommendations. Accurate information regarding healthy weight gain during pregnancy is vital during the prenatal period to prevent long-term morbidity in mother and baby. Our study adds insight into a potential modifiable factor that may contribute to a large proportion of US women gaining an unhealthy amount of weight during pregnancy and the vital role of clinicians and medical organizations in educating and supporting women during this critical window for maternal and child health.

## Appendix

Multimedia Appendix 1 Original queries and query variants.

Multimedia Appendix 2 Top 10 accurate webpages by frequency of appearance during Google searching.

## Abbreviations

ACOG: American Congress of Obstetricians and Gynecologists
BMI: body mass index
IOM: Institute of Medicine
