# Supplementary material for: Crowdsourcing and the Accuracy of Online Information Regarding Weight Gain in Pregnancy: A Descriptive Study
Source: J Med Internet Res. 2016 Apr 7;18(4):e81. doi: 10.2196/jmir.5138 (PMC4840255; doi:10.2196/jmir.5138)
Supplement: Multimedia Appendix 1 [file jmir_v18i4e81_app1.pdf]

Appendix 1: Original Queries and Query Variants

Original Queries	Query Variants
Am I gaining enough weight during pregnancy	am i gaining enough weight during pregnancy
Am I gaining enough weight during pregnancy	how do i know if i am gaining enough weight during pregnancy
Am I gaining enough weight during pregnancy	i am not gaining enough weight during pregnancy
Am I gaining enough weight during pregnancy	what if i am not gaining enough weight during pregnancy
Am I gaining too much weight during pregnancy	am i gaining too much weight during my pregnancy
Am I gaining too much weight during pregnancy	am i gaining too much weight during pregnancy
Am I gaining too much weight during pregnancy	am i gaining too much weight in my pregnancy
Am I gaining too much weight during pregnancy	am i putting on too much weight during pregnancy
Am I gaining too much weight during pregnancy	am i putting on too much weight in pregnancy
Am I gaining too much weight during pregnancy	i feel like i am gaining too much weight during pregnancy
Am I gaining too much weight during pregnancy	what if i am gaining too much weight during pregnancy
Am I gaining too much weight during pregnancy	what should i do if i am gaining too much weight during pregnancy
Fat during pregnancy	back fat during pregnancy
Fat during pregnancy	belly fat during pregnancy
Fat during pregnancy	fat and pregnancy
Fat during pregnancy	fat arms during pregnancy
Fat during pregnancy	fat during pregnancy
Fat during pregnancy	fat face during pregnancy
Fat during pregnancy	fat legs during pregnancy
Fat during pregnancy	fat loss during pregnancy
Fat during pregnancy	fat thighs during pregnancy
Fat during pregnancy	feeling fat during pregnancy
Fat during pregnancy	lose fat during pregnancy
Fat during pregnancy	losing belly fat during pregnancy
Fat during pregnancy	losing body fat during pregnancy
Healthy weight gain during pregnancy	gaining weight fast during pregnancy
Healthy weight gain during pregnancy	healthy weight gain during pregnancy
Healthy weight gain during pregnancy	healthy weight gain during pregnancy australia
Healthy weight gain during pregnancy	healthy weight gain during pregnancy by month
Healthy weight gain during pregnancy	healthy weight gain during pregnancy by week
Healthy weight gain during pregnancy	healthy weight gain during pregnancy chart
Healthy weight gain during pregnancy	healthy weight gain during pregnancy health canada
Healthy weight gain during pregnancy	healthy weight gain during pregnancy if overweight
Healthy weight gain during pregnancy	help gaining weight during pregnancy
Healthy weight gain during pregnancy	rapid weight gain in pregnancy
Healthy weight gain during pregnancy	webmd pregnancy weight gain
How heavy should I be during pregnancy	how heavy should i be during pregnancy
How heavy should I be during pregnancy	how much should i gain weight during pregnancy
How much should I put on during pregnancy	how much should i eat during pregnancy
How much should I put on during pregnancy	how much should i gain weight during pregnancy
How much should I put on during pregnancy	how much should i put on during pregnancy

How much should I put on during pregnancy	how much weight do you put on during pregnancy
How much should I put on during pregnancy	how much weight should i put on during pregnancy
How much should I weigh during pregnancy	how much should a woman weigh during pregnancy
How much should I weigh during pregnancy	how much should i weigh during pregnancy
How much should I weigh during pregnancy	how much should i weigh each week during pregnancy
How much should I weigh during pregnancy	how much should i weigh in pregnancy calculator
How much should I weigh during pregnancy	how much should i weight during pregnancy
How much should I weigh during pregnancy	how much should you weigh during pregnancy
How much should I weigh during pregnancy	how much weight should i weigh during pregnancy
How much weight is too much during pregnancy	how much weight gain is too much during pregnancy
How much weight is too much during pregnancy	how much weight gain is too much in pregnancy
How much weight is too much during pregnancy	how much weight is too little to gain during pregnancy
How much weight is too much during pregnancy	how much weight is too much during pregnancy
How much weight is too much during pregnancy	how much weight is too much in pregnancy
How much weight is too much during pregnancy	how much weight is too much to lose after pregnancy
How much weight is too much during pregnancy	how much weight is too much to lose during pregnancy
How much weight is too much during pregnancy	how much weight loss is too much after pregnancy
How much weight is too much during pregnancy	how much weight loss is too much during pregnancy
Normal weight during pregnancy	healthy weight during pregnancy
Normal weight during pregnancy	healthy weight during pregnancy calculator
Normal weight during pregnancy	healthy weight in pregnancy
Normal weight during pregnancy	normal baby weight during pregnancy
Normal weight during pregnancy	normal weight during pregnancy
Normal weight during pregnancy	normal weight gain during pregnancy by trimester
Normal weight during pregnancy	normal weight gain during pregnancy calculator
Normal weight during pregnancy	normal weight gain during pregnancy month by month
Normal weight during pregnancy	normal weight in pregnancy
Normal weight during pregnancy	weight chart during pregnancy
Normal weight gain during pregnancy	maternity weight gain
Normal weight gain during pregnancy	normal weight gain during pregnancy
Normal weight gain during pregnancy	normal weight gain during pregnancy 20 weeks
Normal weight gain during pregnancy	normal weight gain during pregnancy 25 weeks
Normal weight gain during pregnancy	normal weight gain during pregnancy at 16 weeks
Normal weight gain during pregnancy	normal weight gain during pregnancy by month
Normal weight gain during pregnancy	normal weight gain during pregnancy by trimester
Normal weight gain during pregnancy	normal weight gain during pregnancy chart
Normal weight gain during pregnancy	normal weight gain during pregnancy first trimester
Normal weight gain during pregnancy	normal weight gain during pregnancy for overweight women
Normal weight gain during pregnancy	normal weight gain during pregnancy week by week
Weight during pregnancy	losing weight during first trimester
Weight during pregnancy	losing weight during pregnancy

Weight during pregnancy	not gaining weight during pregnancy
Weight during pregnancy	recommended pregnancy weight gain
Weight during pregnancy	watching weight during pregnancy
Weight during pregnancy	weight during pregnancy
Weight during pregnancy	weight during pregnancy breakdown
Weight during pregnancy	weight during pregnancy calculator
Weight during pregnancy	weight during pregnancy calculator kg
Weight during pregnancy	weight during pregnancy chart
Weight during pregnancy	weight during pregnancy kg
Weight during pregnancy	weight gain during pregnancy calculator
Weight during pregnancy	weight gain during pregnancy chart
Weight during pregnancy	weight loss during pregnancy
Weight during pregnancy	weightlifting in pregnancy
Weight gain per week pregnancy	average weight gain per week during pregnancy
Weight gain per week pregnancy	ideal weight gain per week during pregnancy
Weight gain per week pregnancy	ideal weight gain per week pregnancy
Weight gain per week pregnancy	pregnancy weight gain by week
Weight gain per week pregnancy	pregnancy weight gain distribution
Weight gain per week pregnancy	pregnancy weight gain per week calculator
Weight gain per week pregnancy	recommended weight gain per week pregnancy
Weight gain per week pregnancy	weight gain per week during pregnancy
Weight gain per week pregnancy	weight gain per week pregnancy
Weight gain per week pregnancy	weight gain per week third trimester pregnancy
Weight gain trimester	weight gain each trimester
Weight gain trimester	weight gain first trimester
Weight gain trimester	weight gain third trimester
Weight gain trimester	weight gain third trimester pregnancy
Weight gain trimester	weight gain trimester
Weight gain trimester	weight gain trimester 1
Weight gain trimester	weight gain trimester 3
Weight gain trimester	weight gain trimester breakdown in pregnancy
Weight gain trimester	weight gain trimester pregnancy
