# Supplementary figures and images for: Crowdsourcing and the Accuracy of Online Information Regarding Weight Gain in Pregnancy: A Descriptive Study
Source: J Med Internet Res. 2016 Apr 7;18(4):e81. doi: 10.2196/jmir.5138 (PMC4840255; doi:10.2196/jmir.5138)

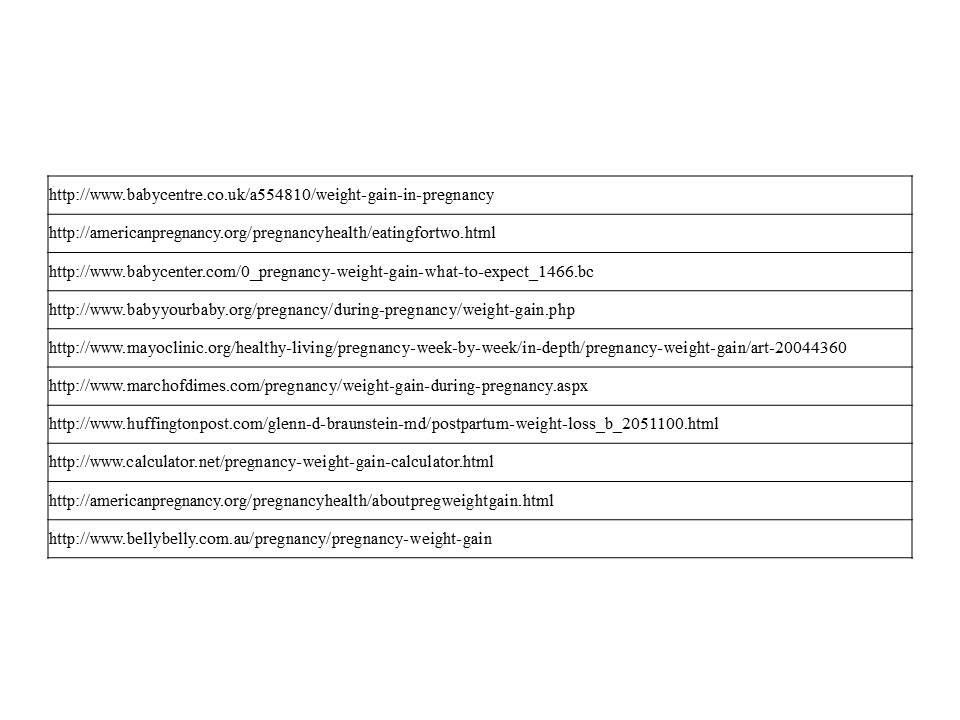

Supplement: Multimedia Appendix 2 [file jmir_v18i4e81_app2.jpg]
